# A model of behavioural response to risk accurately predicts the statistical distribution of COVID-19 infection and reproduction numbers

**DOI:** 10.1038/s41598-023-28752-4

**Published:** 2023-02-10

**Authors:** Fintan Costello, Paul Watts, Rita Howe

**Affiliations:** 1 School of Computer Science, University College Dublin, Dublin, D4, Ireland; 2 Department of Theoretical Physics, National University of Ireland, Maynooth, Ireland; 3 School of Public Health, Physiotherapy and Sports Science, University College Dublin, Dublin, D4, Ireland

**Keywords:** Statistical methods, Computational models, Power law

## Abstract

One clear aspect of behaviour in the COVID-19 pandemic has been people’s focus on, and response to, reported or observed infection numbers in their community. We describe a simple model of infectious disease spread in a pandemic situation where people’s behaviour is influenced by the current risk of infection and where this behavioural response acts homeostatically to return infection risk to a certain preferred level. This homeostatic response is active until approximate herd immunity is reached: in this domain the model predicts that the reproduction rate *R* will be centred around a median of 1, that proportional change in infection numbers will follow the standard Cauchy distribution with location and scale parameters 0 and 1, and that high infection numbers will follow a power-law frequency distribution with exponent 2. To test these predictions we used worldwide COVID-19 data from 1st February 2020 to 30th June 2022 to calculate $95\%$ confidence interval estimates across countries for these *R*, location, scale and exponent parameters. The resulting median *R* estimate was $95\%\; CI=[0.99, 1.01]$ (predicted value 1) the proportional change location estimate was $95\%\; CI=[-0.01, 0.02]$ (predicted value 0), the proportional change scale estimate was $95\%\; CI=[0.99, 1.08]$ (predicted value 1), and the frequency distribution exponent estimate was $95\%\; CI=[1.97, 2.15]$ (predicted value 2); in each case the observed estimate agreed with model predictions.

## Introduction

In simple epidemiological models of disease spread, infection numbers at time *t* are a function of disease transmissibility *p*, incubation rate $\alpha$ and recovery rate $\gamma$ (properties of the disease), of the proportion of infectious and susceptible individuals in the population at time *t*, and of behaviour: in particular, of the average number of contacts individuals make with others at that time. In some models^1^ this contact number is taken as to be constant; in others is treated as a free parameter, varying with time in a way that is not described within the epidemiological model but instead is estimated via fitting the model to data^2–4^ by using mobility or contact tracing datasets^5–7^ or by using assumed seasonal changes in behaviour^8,9^.

It is clear, however, that contact rates between individuals in a population will tend to vary as a function of infection risk, with people reducing contacts and changing behaviour when risk is high in what has been termed a ‘behavioural immune response’^10,11^. Capturing this relationship between human behaviour and infectious diseases is seen as ‘the hard problem of epidemiology’^12^ and a wide variety of behavioural response models have been proposed which link infection numbers and behavioural response in different and often complex ways^13–19^.

A critical problem for research in this area is that of validation: many models are not tested (they give purely theoretical presentations), and when testing is done, it is almost exclusively carried out by fitting the model to existing data; that is, by varying model parameters until model and data agree to some extent^11,12,20^. Such model fits do not act as confirmatory evidence in favour of a model, for at least three reasons. First, a good model fit may arise, not because the model is a useful description of the underlying process, but because parameter variation gives the model flexibility to fit any data. Second, quite different models can often give good fits to the same data; because of this, a good model fit leaves the underlying process unclear. Third, because model fit is specific to both the parameters and the data used, the fact that a model gives a good fit to one specific set of data with a particular choice of parameter values does not imply that this fit will generalise.

Our aim here is to address this problem of validation by presenting a simple and generic behavioural response model (an extension of the standard SEIR compartmental model) and by showing that this model leads to three parameter-free numeric predictions about infection numbers; predictions that, if the model describes the underlying process well, should hold across all sets of data. The first prediction is that the effective reproduction number *R* prior to herd immunity will have a median of 1; the second is that proportional changes in infection numbers will follow the standard Cauchy distribution *C*(0, 1); the third is that the frequency of high infection numbers will follow a power-law distribution $x^{-k}$ with exponent $k=2$. We show that these predictions do, in fact, hold in a large COVID-19 dataset covering 190 countries: the mean estimated *R* value across all countries is statistically indistinguishable from 1, relative changes in new infection numbers follow a standard *C*(0, 1) distribution very closely, and fitting a power law to the frequency distribution for infection numbers for each country, the estimated exponent is statistically indistinguishable from 2.

## ASEIR model of behavioural response to infection risk

Models of behavioural response assume that when people are aware of infection risk, they will change their behaviour (their level of risky contact) with the aim of balancing the risk of infection associated with contact against the various (economic, social, and psychological) gains associated with contact. Our model assumes that each person has a certain constant risk or probability of infection per day, *X*, which they are willing to accept (a level which balances gains from contact with risks from contact), and when they become aware of increased infection risk, they will reduce their number of contacts per day until their overall estimated risk that day is at that level. In a pandemic situation, we expect that awareness will spread as the infection itself spreads, rapidly reaching some saturation level where a large proportion of the population are responding to infection risk. Once this point is reached the probability of infection, and so the overall number of new infections arising in the population, will tend to vary around some constant value or set-point depending on *X* (being pushed away from that point by changes in the infection itself or in various other factors, and being returned to that point by behavioural response to those changes). Assuming that infection confers immunity, this pattern of behavioural response will continue until the total number infected reaches some ‘herd immunity’ level; after this point the number of new infections will necessarily decline irrespective of behavioural changes.

To adjust their behaviour in response to infection risk, people must have some way of estimating risk; as in most behavioural change models, we assume that people estimate the risk of infection at time *t* based on (some approximation of) the number of infections in the population at a previous time $t-L$, where *L* is the lag between an infection occurring and it being known or reported. We express these ideas of homoeostatic behavioural response to risk, spreading awareness of risk, and risk estimation with lag, in an extension of a standard SEIR compartmental model where $S_t$ represents the number of susceptible individuals, $E_t$ the number of exposed individuals (who are incubating infection but not yet infectious), and $I_t$ the number of infectious individuals in a population of size *N* at time *t*, and where $i_t$ represents the number of individuals who were newly infected at that time. Assuming that infection confers lasting immunity, we also have a recovered or removed compartment containing $N- S_t - E_t -I_t$ immune individuals: to avoid confusion with $R_t$, the effective reproduction number at time *t*, we do not refer to this removed compartment here. In this model we have

$$\begin{aligned}S_{t+1}= & {} S_{t}-i_t\\ E_{t+1}= & {} E_{t} + i_t - \alpha E_t\\ I_{t+1}= & {} I_{t} + \alpha E_t - \gamma I_t\end{aligned}$$

(newly infected individuals at time *t* move from the *S* to the *E* compartment, $\alpha E$ individuals move from the *E* to the *I* compartment at time *t*, and $\gamma I$ individuals recover at time *t*). In a standard SEIR model new infection numbers are given by

$$\begin{aligned}i_t = p (K I_t/N) S_t \end{aligned}$$

where the $(K I_t/N)$ term approximates the probability of contact with an infected individual given *K* contacts at time *t* (assuming contacts take place at random) and so $p (K I_t/N)$ gives the probability of a susceptible individual becoming infected given that they make *K* random contacts, and the expected number of new infections is this probability times $S_t$. In our extension of this approach we assume that at time $t=0$ (before the introduction of a new infectious disease), this contact number *K* is set relative to the risk of infection from pre-existing diseases at that time: letting *e* represent the risk of infection from any of those diseases, we then have $eK=X$ (the number of contacts is set so that the risk of infection is approximately *X*) and so $e=X/K$. Susceptible individuals who are not aware of a new infection risk at time $t > 0$ maintain this level of contact *K*, so their risk of infection from the new disease remains at $p K I_t/N$ as in the standard SEIR model. Individuals who are aware of and actively responding to infection risk will adjust their number of contacts $K_t$ so that their estimated probability of infection is, on average, *X*. Let $I_{est}(t)$ represent the *estimated* number of infectious individuals in the population at time *t*. Then for these aware individuals, their total estimated probability of infection from a single contact is

$$\begin{aligned}e+\frac{p I_{est}(t)}{N}= \frac{X}{K}+\frac{p I_{est}(t)}{N}\end{aligned}$$

and so, these individuals will adjust their contacts so that

$$\begin{aligned}\left[ \frac{X}{K}+\frac{p I_{est}(t)}{N}\right] K_t = X \rightarrow K_t = \frac{ X \, K \, N}{XN+p K I_{est}(t)} \end{aligned}$$

Letting $A_t$ represent the number of individuals who are aware of and actively responding to infection risk at time *t*, then since awareness rises when individuals hear about infections among people they know, but falls where there are no such infections, we have

$$\begin{aligned}A_{t+1} = A_t + \left( \frac{ b I_{est}(t)}{N}\right) (N-A_t) - f \left( 1- \frac{ b I_{est}(t)}{N} \right) A_t \end{aligned}$$

where *b* represents the average number of people an individual hears from on a given day and *f* represents the rate at which low risk of infection causes individuals to cease responding. Given this, the average number of contacts at time *t* for the population as a whole is

$$\begin{aligned} \left( 1-\frac{A_t}{N}\right) K+ \left( \frac{A_t}{N}\right) \frac{ X \, K \, N}{XN+p K I_{est}(t)} \end{aligned}$$

The evolution of this ASEIR model depends on the estimated number of infections $I_{est}(t)$. Individuals can only observe or find out about an infection after a certain observation lag *L* (necessarily greater than the incubation time for the infection), and so we simply take $I_{est}(t)=I_{t-L}$ (the estimated number of infections at time *t* is equal to the actual number of infections at time $t-L$), where *L* is within that range. Given this the expected number of new infections at time *t* is

$$\begin{aligned} \begin{aligned} i_t&= \left[ \left( 1-\frac{A_t}{N}\right) K + \left( \frac{A_t}{N}\right) \frac{ X \, K \, N}{XN+p K I_{t-L}} \right] p \,S_t I_t/N \\&= \left[ 1- \left( \frac{A_t}{N}\right) \left( \frac{ p K I_{t-L} }{XN+p K I_{t-L}} \right) \right] p\, K \,S_t I_t/N \end{aligned} \end{aligned}$$

We assume that there is initially no awareness ($A_0=0$). This means that if $b=0$ then $A_t=0$ for all *t*, and so $i_t = p K S_t I_t/N$ and this model includes the standard SEIR approach as a special case. This model depends on 4 parameters from the standard SEIR model (*p*, *K*, $\alpha$ and $\gamma$) and 4 behavioural awareness parameters (*b*, *f*, *X* and *L*). Here we treat *b* as a switching parameter taking on values 0 or *K*, where 0 gives a standard SEIR model while $b = K$ gives an ASEIR model with behavioural response to infection (and where the number of people an individual hears about on a given day is, on average, equal to their number of pre-pandemic contacts).

**Figure 1:**
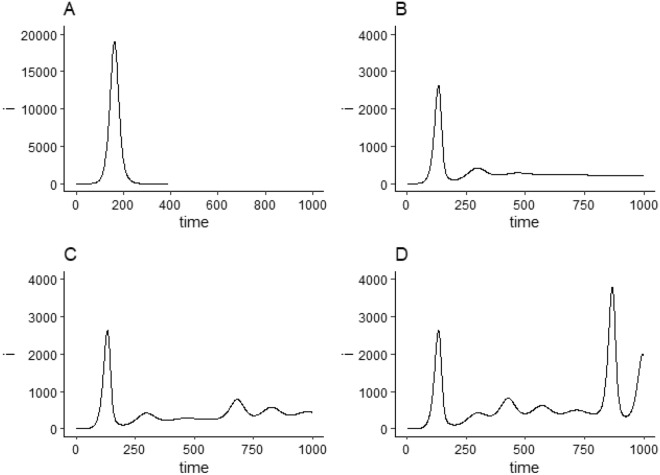
Number of new infections $i_t$ over time generated in simulation runs of the standard SEIR model ($b=0$) and the ASEIR behavioural response model ($b=K$). All simulations with population size $N=10^6$, incubation rate $\alpha =1/10$, recovery rate $\gamma =1/10$, fixed contact number $K=10$ and with behavioural response parameters $x= 1/5000$, $b=K$, $f=1/100$, and lag $L=1/\alpha +1/\gamma = 20$; initial values are $S_0=N$ and $E_0=I_0=A_0=0$. (**A**) SEIR model with a single disease variant with transmission rate and entry time $(p_1=3/100,T_1=1)$. Identical graphs are produced for the SEIR model with 2 or 3 disease variants entering the population (not shown). (**B**) ASEIR model with a single disease variant $(p_1=3/100,T_1=1)$; (**C**) ASEIR model with two variants $(p_1=3/100,T_1=1)$ and $(p_2=7/100,T_2=500)$; (**D**) ASEIR model with three disease variants $(p_1=3/100,T_1=1)$, $(p_2=7/100,T_2=250)$ and $(p_3=25/100,T_2=750)$. In these graphs $t=1$ is the first time-step on which $i \ge 1$ (there is at least one infectious individual in the population), and new infection numbers $i_t < 1$ are not shown. *Note*: data in this figure is illustrative and generated solely by the simulation given the described parameters.

Assuming that *p*, *K* and $\gamma$ are such that the initial reproduction number $R_0=p K/ \gamma > 1$, this model shows a characteristic pattern of evolution in which infection numbers initially rise until awareness *A* reaches a certain level, at which point infection numbers return towards some relatively stable value, with this stability continuing until a point at which herd immunity is reached (after which infection numbers necessarily decline). Figure 1A,B compare the evolution of new infection numbers in a standard SEIR model against their evolution in an ASEIR model (with parameter values selected for demonstrative purposes). Where in the standard SEIR model new infection numbers rise to a high level and then decline to 0, in the ASEIR model infection numbers rise to a much lower level and then tend to oscillate around a constant number of new infections.

The parameters used in the ASEIR model were selected to roughly reflect knowledge about COVID-19 at an order-of-magnitude level: the intention here was not to ‘fit’ data explicitly, but simply to illustrate the ASEIR model’s behaviour with relatively plausible parameter values. Note that the ASEIR model is relatively insensitive to values of these parameters: tests varying $\alpha$, $\gamma$, *K*, *b*, *x* and *f* around these values did not produce any appreciable change in model behaviour. In setting model parameters we took the incubation and infectious periods to be 10 (so that $\alpha =\gamma =1/10$) reflecting the observation that incubation and infectious periods for COVID-19 can fall between 2 and 14 days^21^, and took the lag *L* to be around 20 (assuming that infections are typically reported after the infectious period). We set the average number of contacts per day to be around 5, motivated by contact number estimates^22^ and set *b* (the average number of people an individual hears about on a given day) to the same value. We set the probability of transmission per contact, *p*, for each variant based simply on the assumption that this value was low but increased with each new variant, allowing that variant to spread to dominance (selecting values $p_1=3/100$, $p_2=7/100$, $p_3=25/100$). Parameter *x* (acceptable risk level) was set at 1/5000 based on the observation that infection rates in Ireland tended to stabilise at values somewhere between 200 and 2000 per day: taking 1000 as an order of magnitude estimate, and noting that the population of Ireland is approximately 5 million, this gives an acceptable risk level of 1000 over 5 million, or 1/5000. The forgetting parameter *f* was set at 1/100 on the observation that protests against COVID-19 lockdown or mask measures typically arose around 3–5 months after first introduction of those measures (again, to an order of magnitude): in Ireland, for example, lockdowns were first introduced in March 2020, with first protests in August of that year. Finally, entry times for variants 1, 2 and 3 in Fig. 1D were selected at 0, 250 and 750 to approximately represent the major COVID-19 waves in Ireland, in March 2020, December 2020, and January 2022.

### Modelling disease variants

This stabilisation of new infection numbers assumes no other perturbations or shocks affecting infection numbers. We can extend this simple model to account for such perturbations by considering the emergence of new disease variants, each with different transmission rates *p*. Assuming variants $j \in \{1 \ldots m\}$ each with transmission rate $p_j$ and each entering the population at time $T_j$, we define $E_{j,t}$ and $I_{j,t}$ to be the number of exposed/infectious individuals with variant *j* at time *t* and let

$$\begin{aligned} {\overline{p}}_t = \frac{1}{I_t}\sum _j p_j I_{j,t} \end{aligned}$$

be the weighted average transmission probability at that time. Then we have

$$\begin{aligned} i_{j,t} = \left[ 1- \left( \frac{A_t}{N}\right) \left( \frac{ {\overline{p}}_{t-L} K I_{t-L} }{XN+{\overline{p}}_{t-L} K I_{t-L}} \right) \right] p_{j} K S_t I_{j,t}/N \end{aligned}$$

as the number of new cases of variant *j* at time *t*, and so

**Figure 2:**
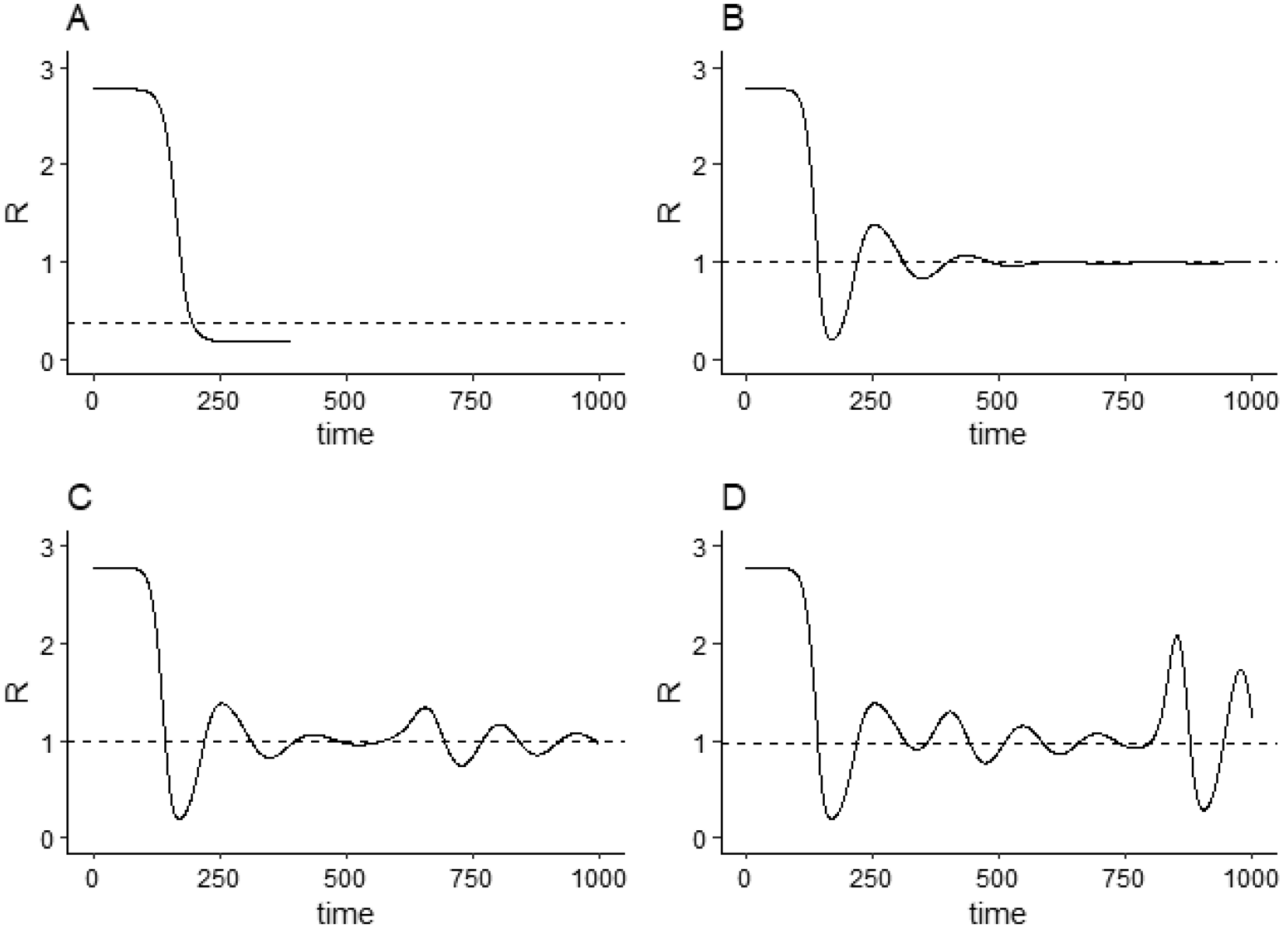
Effective reproduction numbers $R_t$ for the SEIR and ASEIR simulations in figure and 2. Effective reproductive number is calculated from simulated data as $R_t = i_t/(\gamma I_t)$. The dashed line shows the median of the *r* values calculated from $i_t$ and $I_t$ values in each simulation. For the ASEIR simulations, the median $R_t$ was approximately 1; for the SEIR model, the median $R_t$ was 0.18. *Note*: data in this figure is illustrative and generated solely by the simulation given the parameters, see “Modelling disease variants” section.

$$\begin{aligned}{} & {} S_{t+1} = S_{t}-\sum _{j} i_{j,t}\\{} & {} E_{j,t+1} {\left\{ \begin{array}{ll} 1 &{} \textit{ if } t=T_j \\ E_{j,t} + i_{j,t} - \alpha E_{j,t} &{} \textit{ if } t> T_j \end{array}\right. } \\{} & {} E_{t+1} = \sum _{j} E_{j,t+1} \\{} & {} I_{j,t+1} = I_{j,t} + \alpha E_{j,t} - \gamma I_{j,t}\\{} & {} I_{t+1} = \sum _{j} I_{j,t+1}\end{aligned}$$

(and $A_t$ as before).

Figure 1C,D illustrate ASEIR model simulations with the same parameters as before but with 2 or 3 disease variants with different transmission probabilities entering the population at various times. We also ran the SEIR model with these variants: however, the introduction of these variants had no effect on SEIR infection numbers (since herd immunity had been reached by the time these variants entered the population). For the ASEIR model, by contrast, these new variants have a substantial effect on new infection numbers, with ‘plateaus’ in new infection numbers between variant arrival (in Fig. 2C there is a plateau of around 300 new infections per time-step between times $t=300$ and $t=600$, for example).

It is useful to consider the effective reproduction number, $R_t$, produced in these simulations. $R_t$ represents the number of new infections generated by existing infections at time *t*, and can be calculated from simulated data as $R_t = i_t/(\gamma I_t)$; Fig. 2 shows the $R_t$ values calculated at each time-step from the $i_t$ and $I_t$ values generated for each simulation in Fig. 1 (with 3 disease variants for the SEIR model simulation, and 1, 2 and 3 disease variants for the ASEIR model). The median $R_t$ value for the SEIR model is low (around 0.18) while the median $R_t$ value for the three ASEIR simulations are all almost exactly 1 (even when there is significant variability in $R_t$ due to the arrival of disease variants in the population).

A median *R* value of 1 is clearly predicted in the ASEIR model with a single infection (Graph *B* in Figs. 1 and 2) because in that situation behavioural response acts to maintain infection numbers at a relatively constant ‘plateau’ level after the initial wave, necessarily maintaining $R=1$. Similar predictions of $R \sim 1$ arising from such ‘plateaus’ have been made in a number of other models. However, even with multiple infection waves and no plateaus (Graphs *C* and *D*), the ASEIR model still predicts a median *R* value of 1. This more general prediction arises because each individual infection variant, in this model, will be returned to a relatively stable level after its initial wave, and so the total number of new infections (made up of a ‘superposition’ of these individual infection variants) will similarly return to a relatively stable level (until herd immunity is reached).

As these figures illustrate, infection and effective reproduction numbers $R_t$ produced by the ASEIR model have a number of general characteristics: infection numbers do not immediately rise to herd immunity levels and then decline to 0; reproduction numbers, similarly, do not rise and then decline monotonically, but instead vary over time around a median of 1; infection numbers can show various ‘plateaus’ of relatively constant numbers of new infections over long time periods; and both infection and reproduction numbers show noticeable effects of new variants and other stochastic shocks. These characteristics are evident in reported infection and reproduction numbers for the COVID-19 pandemic^15,16,23,24^.

**Figure 3:**
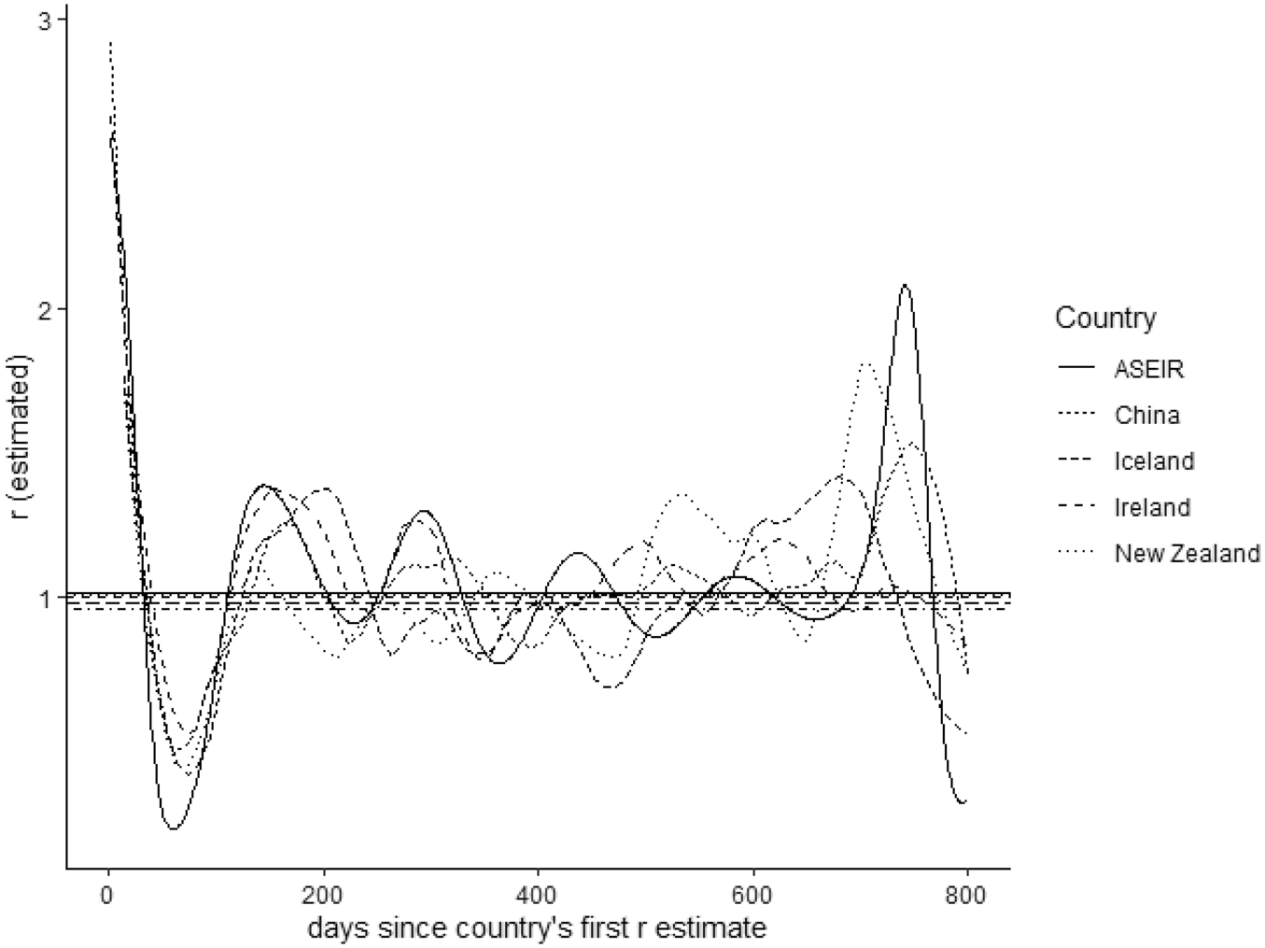
Reported effective reproduction numbers $R_t$ per day for China, Ireland, New Zealand, and Iceland (taken from the OWID COVID dataset and aligned on initial reproduction number; see “Methods”) and reproductive numbers $R_t$ generated by the ASEIR model (from graph D in Fig. 2, aligned by taking day 1 to be $t=110$ in that graph). Horizontal lines show the median $R_t$ value for each country/the model calculated across the entire period (all are almost exactly 1). Model and country $R_t$ values show a common pattern of decline and rebound over the first $\approx 150$ days, and agree closely: in the period up to day 146 (the first ASEIR peak) the Pearson product-moment correlations between ASEIR $R_t$ values and country $R_t$ values were $r=0.81$ (China), $r=0.96$ (Ireland), $r=0.9$ (New Zealand), and $r=0.94$ (Iceland), with all correlations significant at $p < 10^{-15}$.

### Comparison with observed reproduction numbers

The model described above assumes a single initial exposed individual and random homogeneous spread in a single population. As such, the most natural points of comparison are to patterns of infection spread at the origin of a new disease, and to patterns of spread when that new disease enters a comparatively isolated and relatively small population: in these cases the ASEIR model predicts that the same trends in infection and *R* numbers will be seen, at least in the initial period of infection. To test this prediction, we selected 4 countries matching these criteria (the origin country China and 3 island countries selected because they have relatively small populations: Ireland, Iceland, and New Zealand), and compared estimated *R* numbers for COVID-19 in those countries against each other and against *R* numbers produced by the ASEIR model in Fig. 2D. Estimated *R* numbers were taken from the Our World in Data COVID-19 Hub^25^ accessed June 30, 2022 (see ‘Availability of Data and Materials’). The ASEIR model parameters used were the order-of-magnitude estimates described earlier: the model parameters were not changed to fit the data in any way.

Since COVID-19 arrived at different dates in China, Ireland, New Zealand, and Iceland (first *R* estimates for these countries were on 23/01, 25/03, and 27/03 and 05/04/2020, respectively) for comparison purposes we aligned the first *R* number estimate for Ireland, Iceland and New Zealand with the closest reported *R* number for China in the initial phase of the pandemic. $R_t$ numbers produced by the ASEIR model in Fig. 2D were aligned with these numbers by taking day 1 for the ASEIR model to be time $t=110$ in Fig. 2D (the point at which *R* numbers generated by ASEIR begin declining rapidly). Figure 3 shows the aligned *R* values: at the initial stage of infection (up to around 150 days after the first reported $R_t$ number for each country), *R* values for these countries show very similar patterns of steep decline in *R* followed by ‘rebound’ at around $R_t \approx 0.5$, followed by oscillation around approximately 1 in all cases. The ASEIR model follows this pattern closely: over the initial $\approx 150$ day period the correlations between ASEIR $R_t$ values and country $R_t$ values were strong (all $r > 0.8$, all significant at $p < 10^{-15}$).

## Predictions

This fit between predicted and observed $R_t$ values is suggestive, demonstrating as it does that the ASEIR model with the selected parameter values can match the observed evolution of $R_t$ for these countries to at least some degree. This fit does not, however, give support for the behavioural response approach to infectious disease modelling in general; evidential support for a given model is not obtained by fitting a parametrized model to specific data. Instead, evidential support is obtained by testing hypotheses derived from that model which are independent of the model parameters, and which should apply to all observed data, not just to specific fitted data. Because the behavioural response approach assumes that infection and reproduction numbers will return to a given acceptable level and because, like all compartmental models, these are ‘mean field’ predictions (based on and describing expected means, with observed values expected to vary around these means following some error distribution) this approach leads to various predictions about the distributions of $R_t$, $i_t$, and related values.

The ASEIR model’s predictions about the distribution of these values typically hold only in the long run, when infection numbers have reached approximate stability after stochastic shocks (clearly, the model does not predict $R \sim 1$ will hold during an initial infection wave). These predictions also do not hold when herd immunity has been reached (because at that point $R < 1$ necessarily holds independent of any behavioural response). To test these predictions, we must specify the domain where they apply, which we refer to as the ‘oscillatory’ domain.

Assuming that recovery from infection confers lasting immunity, herd immunity is reached at some time *t* where $S_t \approx 1/R_0$, and infection numbers necessarily decrease after that time. In the standard SEIR model, this point is reached relatively quickly, because infection spreads exponentially through the population. In the ASEIR model, this point is reached more slowly: with a single infection and taking *X* to be the acceptable probability of infection, approximately *XN* individuals will be infected per day, and herd immunity will be reached at time $t=h$ where

$$\begin{aligned}N- h X N \approx 1/R_0 \rightarrow h \approx \frac{1}{X}\left( 1-\frac{1}{R_0 N} \right) \approx \frac{1}{X} \end{aligned}$$

We distinguish between the herd immunity and the oscillatory domains by noting a quantitative difference between these two domains: in the herd immunity domain $R_t$ must necessarily remain below 1 (because infection numbers must decrease in this domain), while in the oscillatory domain $R_t$ can go from below 1 to above 1. Taking *h* to be the highest value for which $R_{h-1} \le 1 \le R_{h}$ holds, we see that the region $t \le h$ must be in the oscillatory domain. Similarly, since $R_t$ can go from below 1 to above 1 only after the initial wave of a new variant (or at the start of that initial wave), we see that all variants should have reached approximate stability by time *h*. These ASEIR model predictions are thus expected to hold only in the oscillatory domain $t \le h$ (that is, in the period of time from the start of pandemic infection up to the most recent date at which $R_t$ moved from below to above 1).

### The median value of $R_t$ is 1

We can state the ASEIR model’s specific prediction for *R* as follows: For a given country *c* we take $R_{t,c}$ to be the reproduction number in that country on day *t*. Defining $h_c$ for that country as the most recent day on which $R_{t-1,c} \le 1 \le R_{t,c}$, this model predicts that $R_{t,c}$ will vary around 1 in the oscillatory domain $1 \le t \le h_c$. This prediction holds both in situations where there are clear plateaus in the number of new infections (when these numbers are flat for a long period of time) and also in cases where no such plateaus are observed: in both cases this homoeostatic return is active. Letting $R_{t \le h_c}$ be the median value of *R* in the region $t \le h_c$ for country *c*, random between-country variability means that country medians $R_{t \le h_c}$ will themselves vary across countries around an overall expected mean of 1. More formally, letting $M_1$ represent the mean value of $R_{t \le h_c}$ across a set of different countries, our hypothesis is that the $95\%$ confidence interval for $M_1$ will contain the predicted value 1.

Note that various forms of this general prediction $R_t \sim 1$ have been derived in various behavioural response models and supporting results have been seen in various countries^15,16^ . The main novelty in our proposal is a formal statement of the domain in which this prediction is expected to hold, a formal statistical test of the hypothesis, and a general application of this test to data from all countries worldwide.

### Proportional change in $i_t$ follows Cauchy distribution *C*(0, 1)

In the oscillatory domain infection numbers $i_t$ will tend to vary, in the ASEIR model, in a way that depends on the lag *L* between an infection occurring (at time $t-L$) and that infection being observed by others and causing a behavioural response (at time *t*). This lag is necessarily greater than the incubation period for the infection (an infection becoming observable only after incubation) and means that the observed rate of new infections at time *t* is equal to the actual rate of new infections at time $t-L$. If $i_{t-L} > X$, the acceptable risk level, then the overall behavioural response at time *t* will reduce contact numbers, pushing $i_{t}$ downwards, while if $i_{t-L} < X$ then the overall behavioural response at time *t* will increase contact numbers, pushing $i_{t}$ upwards, and so the difference $i_{t} - i_{t-L}$ varies around 0. Since this overall behavioural response is the sum of all individual responses in the population, from the Central Limit theorem this difference $i_{t} - i_{t-L}$ will follow a Normal distribution $i_{t} - i_{t-L} \sim {\mathcal {N}}( 0, \sigma _{t}^2)$ with some variance $\sigma ^2_{t}$ (which may change over time). The difference $i_{t-2L} - i_{t-L}$ will follow the same distribution (albeit with variance $\sigma _{t-L}^2$). Defining a measure of proportional change in new infection numbers from time $t-2L$ to time *t*,

$$\begin{aligned} D_L(t)= \frac{i_{t} - i_{t-2L}}{i_{t} + i_{t-2L} - 2i_{t-L}}= \frac{(i_{t} - i_{t+L}) - (i_{t-2L} - i_{t-L})}{(i_{t} - i_{t-L}) + (i_{t-2L} - i_{t-L})} \end{aligned}$$

we see that $D_L$ is the ratio of two standard Normal variables (sums of common standard deviations cancelling), and so follows the standard Cauchy distribution *C*(0, 1) (the Cauchy distribution with location parameter 0 and scale parameter 1). The ASEIR model thus predicts that in the oscillatory domain this measure $D_L$ will follow *C*(0, 1) for values of *L* in a region greater than the incubation period of the infection.

We can assess this prediction informally via measures of goodness-of-fit, by asking to what extent the distribution *C*(0, 1) gives a close fit to the distribution of $D_L$ values in the $t \le h_c$ domain. More formally, we note that, if a set of numbers is drawn from some Cauchy distribution *C*, the median of those numbers is an unbiased estimate for the location parameter of *C*, and the median of the absolute values of those numbers is an unbiased estimate for the scale parameter of *C*. Defining $d_c$ to be the median value of $D_L$ for country *c* in the domain $t \le h_c$, and $|d |_c$ to be the median of the absolute values of $D_L$ in that domain, we thus expect that $d_c$ will be distributed around 0 and $|d |_c$ around 1. Letting $M_{2}$ be average value of $d_c$ across a set of different countries and $M_{3}$ be the average value of $|d |_c$ across those countries, our specific hypotheses are that the $95\%$ confidence interval for $M_2$ will contain the predicted location parameter value 0, and that the $95\%$ confidence interval for $M_3$ will contain the predicted scale parameter value 1, for values of *L* in a region greater than the incubation period of the infection.

### Frequency distribution of $i_t$ follows a power law with $k=2$

In the ASEIR model the degree of response to infection risk depends on the degree to which current estimated risk is above the acceptable level *X*: the higher the current value of $I_{est}(t)$, the greater the behavioural response to risk. Here we consider the distribution of values $i_t$ in this model when $I_{est}(t)$ is high: specifically, where

$$\begin{aligned} X \ll p K I_{est}(t)/N\end{aligned}$$

(where the probability of infection given *K* contacts and the estimated number of infections in the population is much greater than the acceptable level of risk, *X*).

In this situation we assume that $A_{t} = A_{t+1} \approx N$ (because infection numbers are high, almost everyone is aware of infection risk); this gives

$$\begin{aligned} \begin{aligned} i_t&= \left[ 1- \left( \frac{ p K I_{est}(t) }{XN+p K I_{est}(t)} \right) \right] p\, K \,S_t I_t/N \\&=\left( \frac{ 1 }{1+\frac{p K I_{est}(t)/N}{X}} \right) p\, K \,S_t I_t/N \\ \end{aligned} \end{aligned}$$

Similarly, in this situation we can assume

$$\begin{aligned}1+\frac{p K I_{est}(t)/N}{X} \approx \frac{p K I_{est}(t)/N}{X} \end{aligned}$$

giving

$$\begin{aligned} \begin{aligned} i_t&\approx \left[ \frac{ X }{p K I_{est}(t)/N} \right] p\, K \,S_t I_t/N = X S_t \left( \frac{I_t}{ I_{est}(t)} \right) \\ \end{aligned} \end{aligned}$$

Finally, assuming that estimated infection numbers are to some degree realistic (that the ratio $I_t/ I_{est}(t)$ varies around 1) we can approximate the change in values of *i* at time *t* as

$$\begin{aligned}i_{t+1} - i_{t} \approx X \left[ S_{t+1} - S_t \right] = - X i_{t} \end{aligned}$$

and the rate of change in *i* at time *t* is proportional to the value of *i* at that time. In the continuous case, this corresponds to

$$\begin{aligned}\frac{dt}{di} = - \frac{1}{X i}\end{aligned}$$

and given that some number of infections *i* has occurred, the amount of time infection numbers will remain in some region $\Delta$ around *i* will be proportional to $\Delta /i$. Assuming that infection numbers are ‘measured’ at some constant rate and the true infection number has reached a value *i* some time since the last measurement, this means that the probability of obtaining a measured infection number in the region $\Delta$ around *i* will also be proportional to $\Delta /i$; in other words, the conditional probability of recording a new infection count in the region $\Delta$ around *i* (given that there were *i* new infections at some time since the last measurement) is expected to follow a power law $p(i) \sim i^{-\lambda }$ with exponent $\lambda =1$.

Given some fixed bin size $\Delta$, let $n_j$ be the number of recorded new infection counts *i* that fall into bin *j* (that is, where $\Delta j < i \le \Delta (j+1)$). Similarly, let $\phi _{\Delta }$ be the frequency distribution for values $n_j > 0$, so that $\phi _{\Delta }(n)$ gives the number of bins for which $n_j=n$. Since infection numbers in each bin in this distribution have occurred at least once, the probability of observing an infection *i* that falls into any one of these bins is approximated by the conditional probability $p(i) \sim i^{-\lambda }$ with $\lambda =1$. If a variable’s probability distribution follows a power law with exponent $\lambda$, then the associated frequency distribution $\phi$ will follow a power law with exponent $k=1+1/\lambda$^26,27^; since probabilities *p*(*i*) for these bins follow a power law with exponent $\lambda =1$, our prediction is the frequency distribution $\phi _{\Delta }$ will follow a power law with exponent $k=1+1/\lambda =2$. Note that, unlike our predictions about *R* and $D_L$ (both of which describe long-run oscillatory behaviour and so are limited to the oscillatory domain), this power-law prediction is focused on the tail of high infection numbers (primarily caused by the arrival of new variants in the population), and thus holds generally, and not just in the oscillatory domain.

As before, this prediction can be assessed informally via measures of goodness-of-fit: by asking to what extent the frequency distribution for *i* (given a certain value of $\Delta$) is fit by a power-law distribution with exponent 2. More formally, fitting a general power law to the frequency distribution of *i* for a given country *c* and letting $k_c$ be the best-fitting exponent value obtained for that country and then taking $M_4$ to be mean value of $k_c$ across a set of different countries, our hypothesis is that the $95\%$ confidence interval for $M_4$ will contain the predicted value 2.

## Methods

We tested these predictions about $M_1$, $M_2$, $M_3$ and $M_4$ using publicly available data from the Our World in Data COVID hub^25^ for the period from the start of the pandemic up to June 30, 2022 (see ‘Availability of Data and Materials’). This dataset gives the number of new COVID-19 infections reported each day for 231 countries under the variable name *new cases*, from the Johns Hopkins University COVID-19 Data Repository^28^, and a smoothed version of this measure under the variable name *new cases smoothed* (alongside a population-normalised measure *new cases smoothed per million*). This dataset also gives the estimated reproduction number each day under the variable name *reproduction rate*, with estimation carried out using a Kalman Filter approach^23^. Some countries in the dataset had no values associated with one or more of these variables on any day: we cleaned the dataset by removing all such countries, leaving data from 190 countries for analysis.

One problem with the OWID COVID-19 data arises because countries frequently reported 0 new case numbers on certain days: around $25\%$ of new case numbers reported in the OWID dataset were 0, with many countries having reliable patterns of 0 new case numbers on weekend days only. These 0 values clearly do not reflect a change in infection numbers; instead, they simply indicate gaps in reporting. Derived measures such as reproduction rate and smoothed new case numbers are calculated from these reported values and so are similarly affected by these gaps, but to a lesser degree. In an attempt to avoid these gaps in our analysis we further clean the dataset by excluding, for each country, any day with 0 new case numbers reported for that country. Our analysis thus considers the evolution of infection numbers over consecutive reporting days, with reporting gaps removed.

For a given country *c* and day *t* we take $R_{t,c}$ to represent the value of the OWID *reproduction rate* variable for that country on that day. Taking $h_c$ to be the highest value (the latest day) for which $R_{h-1,c} \le 1 \le R_{h,c}$ holds for country *c*, the oscillatory domain for that country is $t \le h_c$ and our predictions concern the value and confidence intervals for $M_1$, $M_2$ and $M_3$ calculated from the cleaned dataset in that domain. There were 6 countries where $R_{t,c} < 1$ held for all reported days: these countries were excluded from analysis of oscillatory domain results, leaving 184 countries giving oscillatory domain data.

In assessing predictions about $M_2$ (the mean proportional change in new infection numbers, $d_c$) and $M_3$ (the mean of the absolute value of that change, $|d |_c$) we take $i_t$ for a given country *c* to represent the OWID variable *new cases* for that country, and calculate $d_c$ and $|d |_c$ from values of this variable in the oscillatory domain. Note that, while the OWID variable *new cases smoothed* gives a more accurate estimate of new infection numbers at a given time (because the smoothing process reduces the variability caused by reporting gaps), we cannot use this smoothed variable to assess the distribution of proportional changes in new infection numbers over time (because the smoothing process itself removes some proportional changes from the data, and so systematically alters this distribution).

In assessing our prediction about $M_4$ (the mean estimated power-law exponent $k_c$), we take $i_t$ for a given country *c* to represent the OWID variable *smoothed new cases* for that country, and calculate $k_c$ from the frequency distribution of this variable in the entire dataset (not just the oscillatory domain). We use the *new cases smoothed* variable for this analysis because that variable gives a more accurate estimate of new infection numbers, and because the smoothing process has no particular effect on exponent estimation.

Since we do not know the statistical distributions for values of interest $M_1$,$M_2$,$M_3$ and $M_4$, we estimate confidence intervals for these values using both the assumption of normally distributed error (via a *t*-test) and using a standard non-parametric bootstrapping method. Our analysis of the power-law prediction $M_4$ applies to frequency data, which is produced by placing numbers $i_{t}$ into bins of a certain size $\Delta$. A central problem for such frequency analysis arises with the choice of bin size $\Delta$: different choices for $\Delta$ will produce different numbers of bins for a given set of infection numbers $i_{t}$, making the resulting frequency data easier or harder to fit (depending on whether the number of bins obtained is small or large). We deal with this problem by automatically setting a bin size $\Delta _c$ for each country so that each country’s infection number data falls into the same number of bins *B*, where *B* is the largest number such that every country’s data can be placed into at least *B* distinct bins. This procedure ensures that power-law fits to frequency data for different countries are not affected by artefacts arising from the choice of bin size.

**Figure 4:**
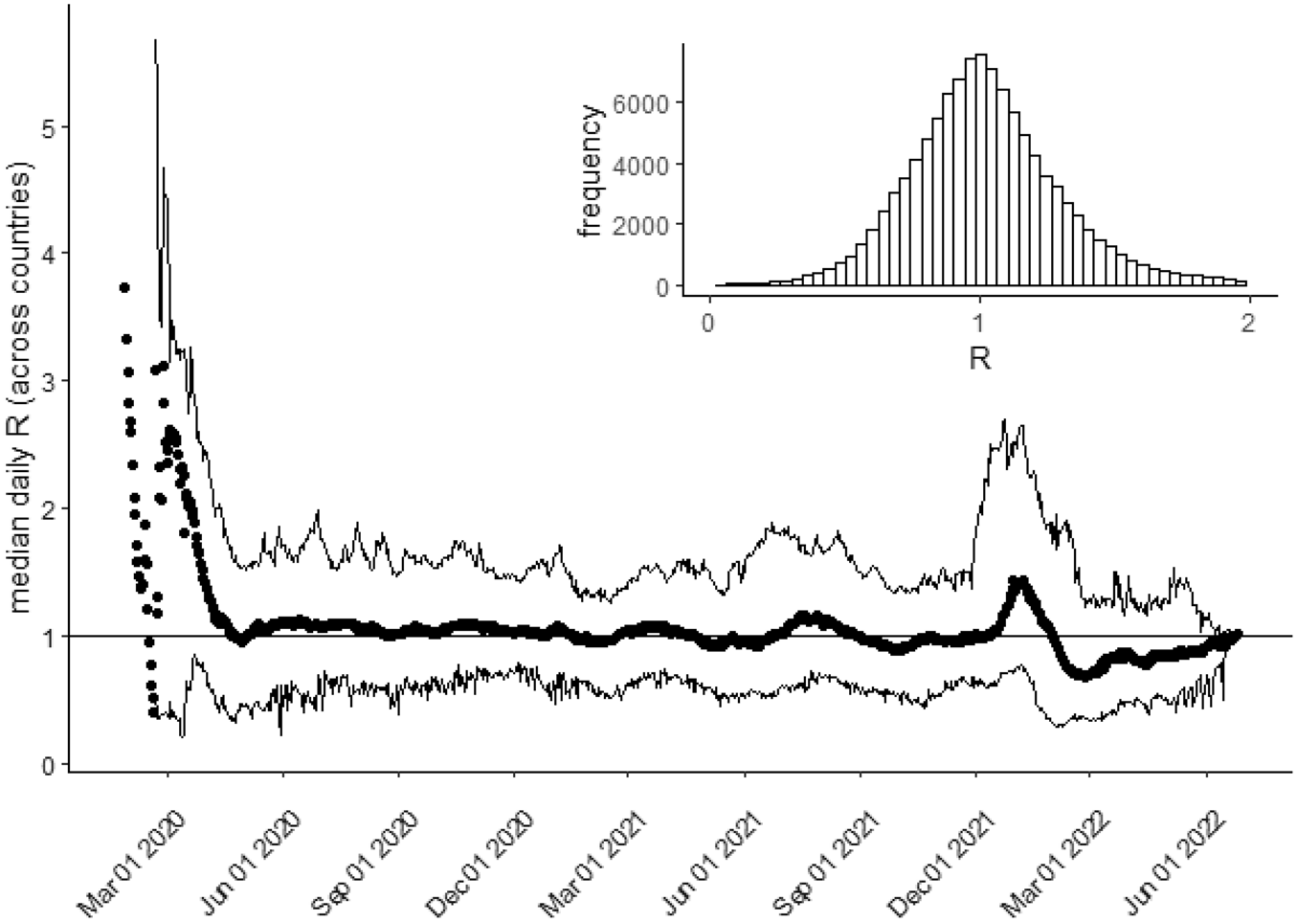
Median of *R* values on each day (points) with $2.5\%$ and $97.5\%$ quantiles for country *R* values on each day (lines). The inset shows a histogram of *R* values (bin size 0.1). There are no quantiles before 21 February 2020, because only *R* values for China are reported before that date. *Data source*: OWID COVID dataset, see “Data availability” section for details.

All statistical and modelling analysis was carried out RStudio using R version 4.0.5^29^, using packages data.table, ggplot2, lubridate and patchwork for general analysis and graphing^30–33^ and using packages qqplotr^34^ for quantile-quantile plots, nptest^35^ for non-parametric confidence intervals; and poweRlaw^36^ for power-law fits. A complete R script that implements the ASEIR model, downloads the OWID COVID-19 data, carries out all statistical and data analysis, and generates all figures reported here is available online (see ‘Availability of Data and Materials’).

## Results

Figure 4 shows the distribution of *R* values for countries in the OWID dataset on each day *t* in the oscillatory region, with a histogram showing the frequency of individual *R* values. Both show *R* centred around 1, consistent with our first prediction.

**Figure 5:**
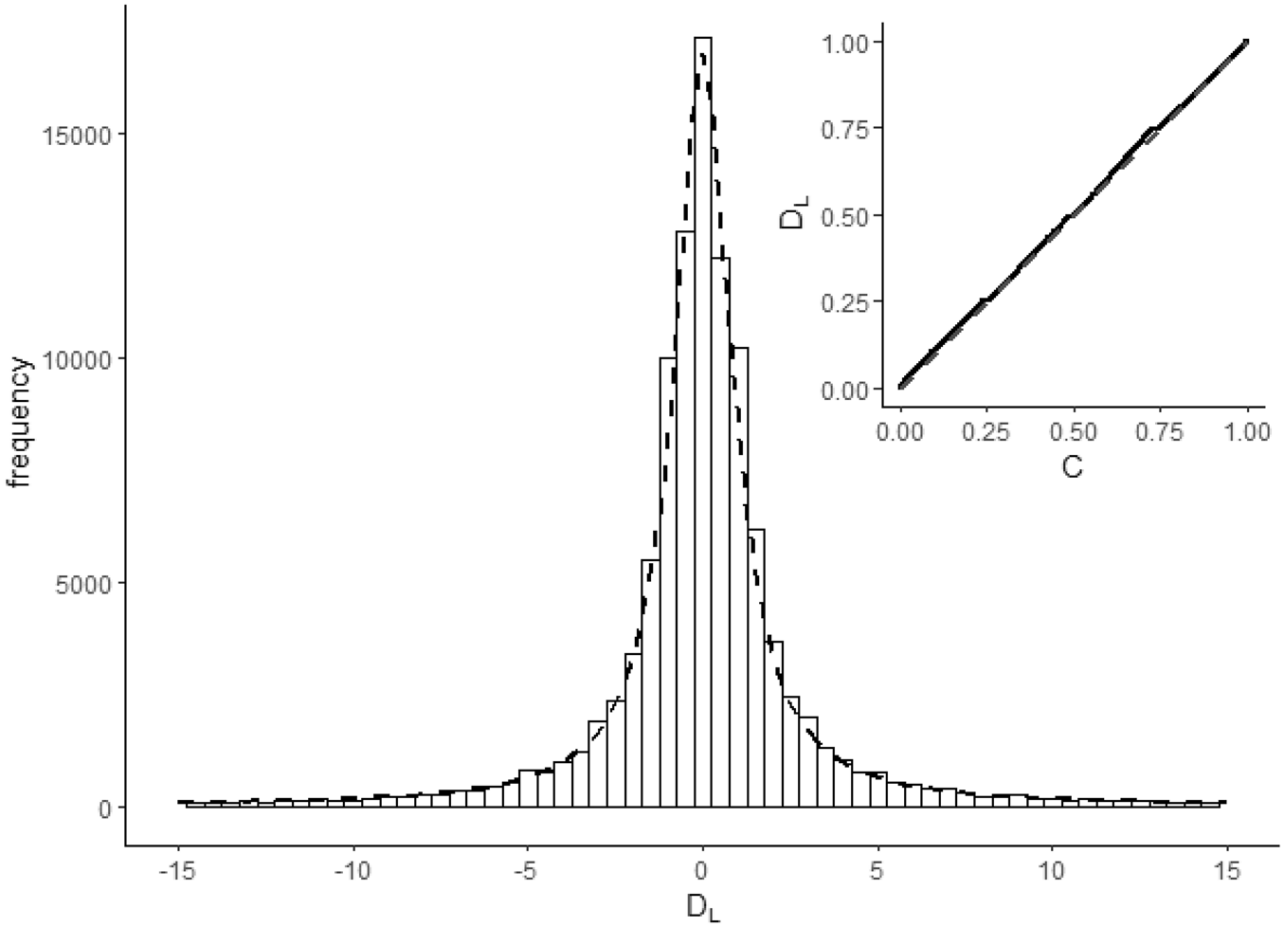
Histogram of $D_L$ for $L=7$ calculated from the cleaned dataset in the central $-15\ldots 15$ range (bin size 0.5) with standard Cauchy distribution *C*(0, 1) (dashed line, *C* distribution scaled by bin size and total histogram frequency for comparison). The inset shows a probability-probability plot comparing theoretical and observed cumulative probabilities across the entire range: the solid line in that plot is actually made up of over 50, 000 points, one for each $D_L$ value calculated in the dataset for $L=7$: the dashed diagonal line (mostly hidden by these points) is the line of identity between theoretical and observed cumulative probabilities. *Data source*: OWID COVID dataset, see “Data availability” for details.

To test this $R \sim 1$ prediction formally, we calculated the median $R_{t \le h_c}$ value for each country *c* in the OWID dataset across the COVID-19 pandemic. The overall mean of these values was $M_1 = 1.0$ with a $95\%$ confidence interval for the mean of $0.99 \ldots 1.01$ both when calculated via a *t*-test ($t=-0.34, df = 183, p = 0.73$) and via the non-parametric bootstrap estimate, confirming the prediction.

To assess our $D_L$ predictions informally we compare $D_L$ values calculated from the cleaned OWID dataset against the theoretical distribution *C*(0, 1). For each country, we calculated $D_L(t)$ for every day in the dataset using the OWID *new cases* variable in that country’s oscillatory region, for values of *L* from 7 (assuming the incubation period for COVID-19 is approximately 6) to 30 (assuming that the reporting of case numbers will not take more than one month). For some days $D_L(t)$ could not be calculated because one of the component infection numbers was missing or resulted in division by 0; these values of $D_L(t)$ were dropped from analysis. Figure 5 (inset) shows a probability-probability plot comparing the cumulative probability of $D_L$ for $L=7$ against that of *C*. Correlation of cumulative probabilities is a measure of goodness of fit between observed and theoretical values; here the correlation was high ($r=0.999$). Since probability-probability plots overweight extreme values, we also analysed the relationship between *C*(0, 1) and $D_L$ for values near the midpoint of the range, by selecting the subset of $D_L$ values between $-15$ and 15 ( $93\%$ of the total sample). Figure 5 (main) shows a histogram of these values. The correlation between $D_L$ and *C* values for this central-region histogram was $r=0.99$. Similar results held for other values of *L*.

To test prediction $M_2$ formally we obtained, for each country *c*, location estimates $d_c$ for values of *L* from 7 to 30 by calculating the median value of $D_L$ for that country for each value of *L*, and setting $M_2(c)$ to be the mean of these location estimates for that country across all values *L*. We took $M_2$ to be the average of these $M_2(c)$ values across all countries. The overall mean of these values was $M_2 = 0.01$ with a $95\%$ confidence interval for the mean of $-0.01 \ldots 0.02$ when calculated via a *t*-test ($t = 1.15, df = 183, p = 0.25$) and the same confidence interval when calculated via the non-parametric bootstrap estimate. This confirms prediction 2.

We similarly obtained, for each country *c*, scale estimates $|d |_c$ for values of *L* from 7 to 30, by calculating the median of the absolute value of $D_L$ for each value of *L*, and setting $M_3(c)$ to be the average of these scale estimates. The overall mean of these values was $M_3 = 1.08$ with a $95\%$ confidence interval for the mean of $1.04 \ldots 1.1$ in both *t*-test and non-parametric analysis. While this is very close to the predicted scale estimate of $M_3=1.0$, the predicted value falls outside the calculated confidence interval, and so prediction $M_3$ is not confirmed.

The fact that the estimated scale parameter here is marginally higher than the predicted value (1.08 versus 1) could arise as a consequence of overextension of the oscillatory region for some countries: if the identified oscillatory region bound $h_c$ for country *c* in fact included the initial rising section of an infection wave, values $D_L$ in that region will be biased upwards by that wave, producing an increase in the scale estimate. As a post-hoc test of this proposal, we calculated for each country the number of days in the dataset outside the oscillatory region (days where $t > h_c$) and re-ran our analysis excluding any countries where this number of days was small. Excluding all countries where $|t > h_c |\le 10$ give $M_3 = 1.04$ with a $95\%$ confidence interval for the mean of $0.99 \ldots 1.08$, supporting prediction $M_3$.

**Figure 6:**
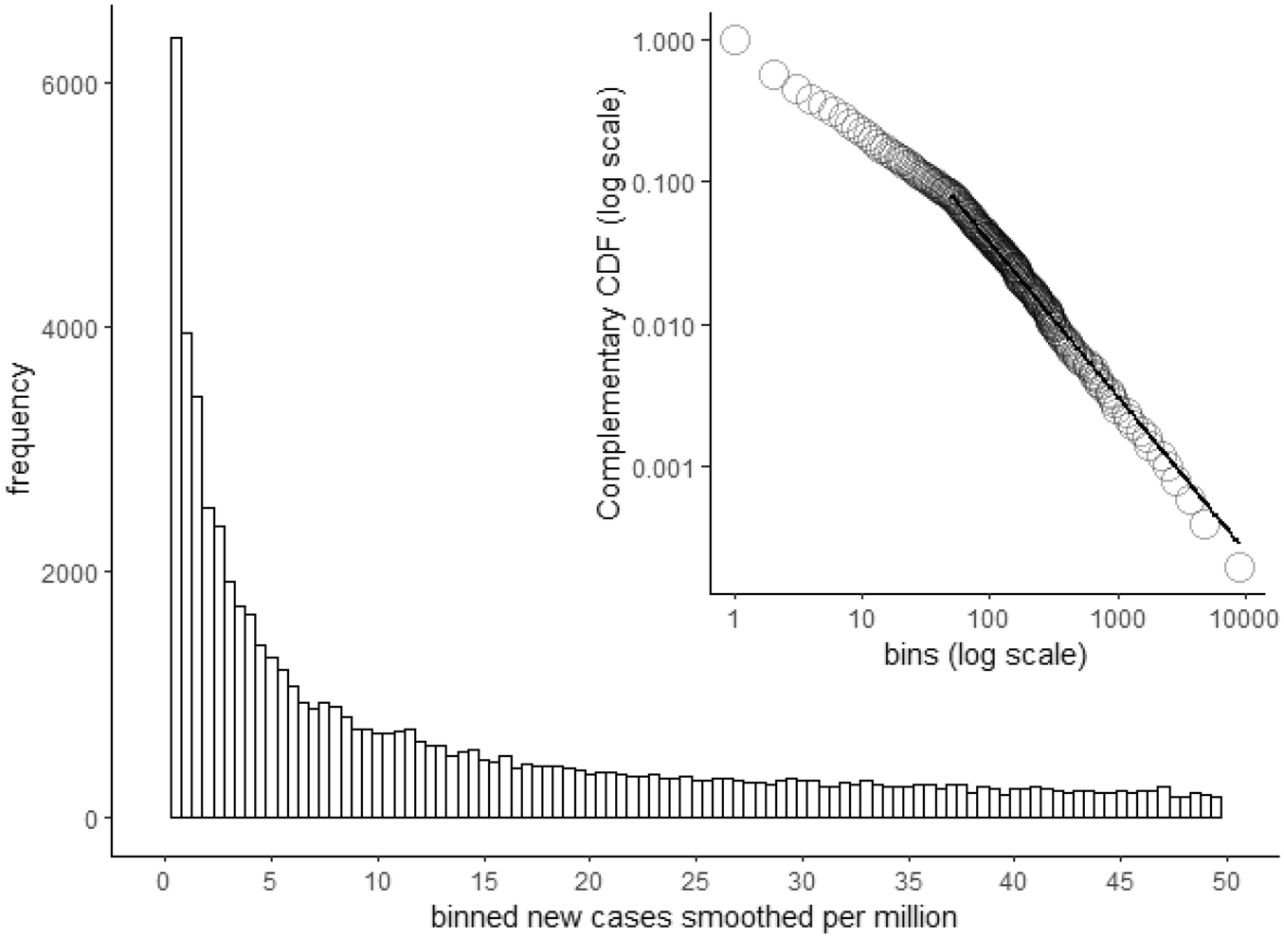
Histogram showing the frequency of smoothed new cases per million (bin size 0.5) across all countries in the OWID dataset. For illustrative purposes only the first 50 frequency bins are shown. The inset shows a plot of complementary cumulative probability (CCDF) across all bins: the solid line shows the theoretical CCDF value for the best fitting power law for this frequency data, with $k=2.08$ and $x_{min}=49$. The agreement between the solid line and the CCDF data points gives an informal illustration of the power-law fit.

To test our power-law prediction $M_4$ informally, we produced a frequency table of smoothed new cases per million across all countries in the OWID dataset, with a bin size of 0.5 (Fig. 6). We found the best-fitting power law for this frequency data using the R powerLaw package^37^. When fitting a power law to data, it is usually argued that only the tails of the distribution (greater than some value $x_{min}$) follow a power law; this assumption is explicit in the behavioural response account, where a power law is assumed to hold only for high new infection numbers. The powerLaw package returns the best-fitting $x_{min}$ and *k* values for the given data; for the OWID data, the best fit was obtained with $k=2.08$ and $x_{min}=49$ (new infection numbers greater than 49 per million are best fit by a power law with $k \approx 2$). Figure 6 plots the frequency of smoothed new cases per million across all countries in the OWID dataset in the first 50 of these bins. A standard way to assess power-law fits informally is via comparison of observed and theoretical ‘complementary cumulative distribution functions’ or CCDFs^38^; the inset in Fig. 6 plots the observed CCDF versus the theoretical CCDF predicted for this value of *k*. Note that the theoretical CCDF (solid line) starts at $x_{min}=49$, and that there is a noticeable ‘turn’ in the observed CCDF at that point. In the context of the ASEIR model, this point represents a transition to the ‘high infection numbers’ domain.

To test prediction $M_4$ formally, we first obtained, each country in the cleaned OWID dataset, the number $B_c$ equal to the largest integer such that the full set of *i* data for country *c* can be placed into $B_c$ equal-sized bins. We then set *B* equal to the minimum value of $B_c$ across all countries, so that *B* is the largest number such that every country’s data can be placed into at least *B* distinct bins. Given this *B* we then obtained, for each country *c*, the largest bin size $\Delta _c$ such that country *c*’s data will be placed into *B* bins and using that bin size $\Delta _c$ produced a frequency table of smoothed new case numbers for that country. For each country we used the powerLaw package to find the best-fitting power law for that country’s frequency table. Letting $k_c$ be the best-fitting power law exponent for country *c*, we took $M_4$ to be the mean value of $k_c$ across all countries. The overall mean of these values was $M_4 = 2.06$ with a $95\%$ confidence interval for the mean of $1.97 \ldots 2.15$ when calculated via a *t*-test ($t = 1.33, df = 189, p = 0.18$) and via the non-parametric bootstrap estimate. This confirms prediction 4.

## Discussion and conclusions

In this paper we have presented an extension of the standard SEIR compartmental model of infection to include spreading awareness of and behavioural response to infection risk. We have shown that this model can naturally account for the effect of various disease variants arriving in a population over time and matches initial patterns of rapid decline and rebound in reproduction numbers for the COVID-19 pandemic for selected countries. To validate this model, we derive various parameter-free numeric predictions from this approach; analysis of COVID-19 data at both aggregate (world) and individual country levels gives explicit confirmation for these predictions, validating the behavioural response approach to modelling infection spread, and demonstrating some striking statistical regularities in the distribution of infection numbers.

It is useful to specify the situations in which we expect these statistical regularities to hold. First, these results assume that a large proportion of the population will become aware of and respond to the risk of infection, and so apply to epidemic or pandemic situations only: we do not expect this model to describe infection spread in narrower outbreak situations. Second, this model depends on the assumption that people’s estimates of infection risk at time *t* will reflect the number of new infections at some recent time $t-L$. This assumption holds for infections with short incubation and recovery periods; for infections where these periods are longer, this assumption does not hold. Third: this model makes the simplifying assumption that people are free to limit their number of contacts to match their acceptable level of risk. For some demographics this is not the case: people in poverty, for example, may be economically unable to limit their contacts in this way, and so will have an estimated risk of infection systematically above their acceptable risk level. Assuming that people’s acceptable risk levels are well-calibrated, this predicts increased infections in such demographics relative to the population as a whole^39,40^. Letting $K_{min}$ represent the lowest possible average contact rate for the population as a whole given these constraints on contact numbers, then $R_{min}=p K_{min}/ \gamma$ is the minimum possible reproduction number, and if $R_{min} > 1$ then the disease will spread exponentially through the population irrespective of behavioural response; while if $R_{min} < 1$ then behavioural response will act to maintain $R \sim 1$ in the oscillatory period of the infection.

The model makes a number of other simplifying assumptions: no vaccination, perfect and lasting immunity after infection, no quarantining or reduction of contact numbers among infected individuals. More realistic (and so more complex) versions of the model can be constructed to include vaccination, waning immunity, and quarantine responses. However, the statistical regularities described above will necessarily hold in these more complex models just as in the simple model described above. This is because while vaccination, waning immunity, and quarantine all have clear effects on infection risk, in the ASEIR model behavioural response to this risk will continue to act to maintain $R \sim 1$ (with increased vaccination numbers, for example, causing a reduction in both perceived infection risk and in infection numbers, and this reduction in risk causing a corresponding increase in contact numbers and so a subsequent rise in infections, thus maintaining *R* around 1). The effect of vaccination, in these more complex models, is to shorten the oscillatory period and increase progress towards herd immunity, while the effect of waning immunity is to lengthen the oscillatory period and postpone herd immunity. An important aim for future research is to test these predictions about the effects of vaccination programs and of reinfection rates against data on COVID-19.

## Supplementary Information

Supplementary Information.

## Data Availability

All data used in this analysis is publicly available online from the Our World In Data COVID hub https://ourworldindata.org/coronavirus in the combined data file https://raw.githubusercontent.com/owid/covid-19-data/master/public/data/owid-covid-data.csv. R code implementing the ASEIR model, downloading this data file and running all analyses is publicly available online from the Open Science Foundation repository https://osf.io/29ayn/.

## Code availability

All code used for analysis is included as a supplementary file.
